# Water Selective Imaging and bSSFP Banding Artifact Correction in Humans and Small Animals at 3T and 7T, Respectively

**DOI:** 10.1371/journal.pone.0139249

**Published:** 2015-10-01

**Authors:** Emeline J. Ribot, Didier Wecker, Aurélien J. Trotier, Benjamin Dallaudière, William Lefrançois, Eric Thiaudière, Jean-Michel Franconi, Sylvain Miraux

**Affiliations:** 1 Centre de Résonance Magnétique des Systèmes Biologiques, UMR 5536, CNRS/University Bordeaux, Bordeaux, France; 2 Bruker Biospin MRI GMBH, Ettlingen, Germany; Shenzhen institutes of advanced technology, CHINA

## Abstract

**Introduction:**

The purpose of this paper is to develop an easy method to generate both fat signal and banding artifact free 3D balanced Steady State Free Precession (bSSFP) images at high magnetic field.

**Methods:**

In order to suppress fat signal and bSSFP banding artifacts, two or four images were acquired with the excitation frequency of the water-selective binomial radiofrequency pulse set On Resonance or shifted by a maximum of 3/4TR. Mice and human volunteers were imaged at 7T and 3T, respectively to perform whole-body and musculoskeletal imaging. “Sum-Of-Square” reconstruction was performed and combined or not with parallel imaging.

**Results:**

The frequency selectivity of 1-2-3-2-1 or 1-3-3-1 binomial pulses was preserved after (3/4TR) frequency shifting. Consequently, whole body small animal 3D imaging was performed at 7T and enabled visualization of small structures within adipose tissue like lymph nodes. In parallel, this method allowed 3D musculoskeletal imaging in humans with high spatial resolution at 3T. The combination with parallel imaging allowed the acquisition of knee images with ~500μm resolution images in less than 2min. In addition, ankles, full head coverage and legs of volunteers were imaged, demonstrating the possible application of the method also for large FOV.

**Conclusion:**

In conclusion, this robust method can be applied in small animals and humans at high magnetic fields. The high SNR and tissue contrast obtained in short acquisition times allows to prescribe bSSFP sequence for several preclinical and clinical applications.

## Introduction

Balanced Steady State Free Precession sequence (bSSFP) is increasingly employed in clinical practice due to the high Signal-to-Noise Ratio (SNR) generated in short acquisition times. The bSSFP sequence is usually performed for cardiac imaging, spinal cord imaging and musculoskeletal imaging [1]. However, a major disadvantage is the presence of banding artifacts that occur at locations in the image where the main field inhomogeneity is a multiple of 1/TR Hz, producing repetitive signal loss on the images. Alternating phase radiofrequency (RF) pulse acquisition technique implying the acquisition of multiple images, has been combined with the maximum-intensity, the complex-sum, the magnitude-sum combinations to eliminate them. However, the combination with the “Sum-Of-Square” (SOS) reconstruction has been shown to be the most robust approach without affecting the SNR or the contrast generated by the bSSFP sequence for human knee and brain imaging [2]. This enabled tumor assessment in mice at 1.5T [3,4] and at high magnetic fields [5,6]. Even though this technique is reliable, two main issues remain: the amount of banding artifacts increases with the magnetic field strength due to an increase in field heterogeneity and susceptibility effects [5] and, the longer the TR, the more difficult it is to correct banding artifacts. Another difficulty is the high SNR from adipose tissue due to T2/T1 contrast engendered by the sequence. Fluid, bone-marrow, sub-cutaneous and visceral fat exhibit similar hyperintense MR signals, limiting the detection of synovial fluid and cartilage at the knee joint, bone-marrow edema and lesions or metastases developing in lymph nodes. Furthermore, the presence of fat signal generates a chemical shift artifact, that can affect the interpretation of the images and alter the accuracy of volume measurements. To suppress fat signal from bSSFP images, several techniques have been developed including Dixon technique [7], Alternating-TR technique [8], Fluctuating Equilibrium MR-bSSFP [9,10], Linear Combination technique [11], multiple-TR [12], phase-sensitive bSSFP [13,14], oscillating bSSFP [15], variable flip angles [16] or by inserting magnetization preparation [17]. However, all of these techniques induce restrictions in TE/TR values and SAR, and increase sensitivity to field heterogeneity and RF nonlinearity.

On the other hand, water or fat selective imaging is commonly performed with a spectrally-selective binomial RF pulse to excite protons in a specific resonance frequency range [18,19]. Even though no TR value restriction and less sensitivity to B1 inhomogeneities are encountered, this technique has been combined with bSSFP sequence mostly at 1.5T to limit the presence of banding artifacts [20,21]. Imaging at higher magnetic field can be beneficial to shorten acquisition time due to higher SNR, while more susceptibility effects are expected, that would deteriorate image quality.

Consequently, very few studies have attempted to simultaneously reduce fat signal and banding artifacts and exclusively at 1.5T [13,22]. Only a very recent study developed a 2-point Dixon bSSFP sequence combined with Complex-Sum reconstruction to suppress banding artifact at 3T [23]. However, restrictions in TE values limit the applications of this method.

Thus, the goal of our study was to demonstrate that 3D bSSFP can be combined with both the method of frequency offset and water-selective excitation to generate images free of banding artifact and with reduced fat signal in the entire FOV at high magnetic fields. Furthermore, as acceleration techniques are routinely performed in clinical settings, another objective of our study was to evaluate that the new method is compatible with parallel imaging. Indeed, most of the MR reception coils are now arrays of multiple surface coils in order to improve SNR and decrease acquisition time.

After theoretical considerations, this method was applied at 7T in mice and also at 3T for human studies for several applications.

## Theory

### Binomial pulses

The excitation pulses were designed to contain several sub-pulses with binomial amplitude ratios. Simulations were performed to determine the optimal parameters (sub-pulse intensity and duration, interpulse duration representing the gap duration between each sub-pulses) of the binomial pulse that would induce a high frequency selectivity while maintaining the pulse duration as short as possible. Two highly frequency-selective binomial pulses were thus designed: a 5 hermite-shaped sub-pulses binomial pulse (1-2-3-2-1 scheme; interpulse duration = 200μs; sub-pulse duration = 102.4μs; total length = 1.3ms) at 7T and a 4 hermite-shaped sub-pulses binomial pulse (1-3-3-1 scheme; interpulse duration = 730μs; sub-pulse duration = 200μs; total length = 2.9ms) at 3T. The maximum amount of sub-pulses that could be set at 3T was restricted to 4 by the constructor.

**Fig 1:**
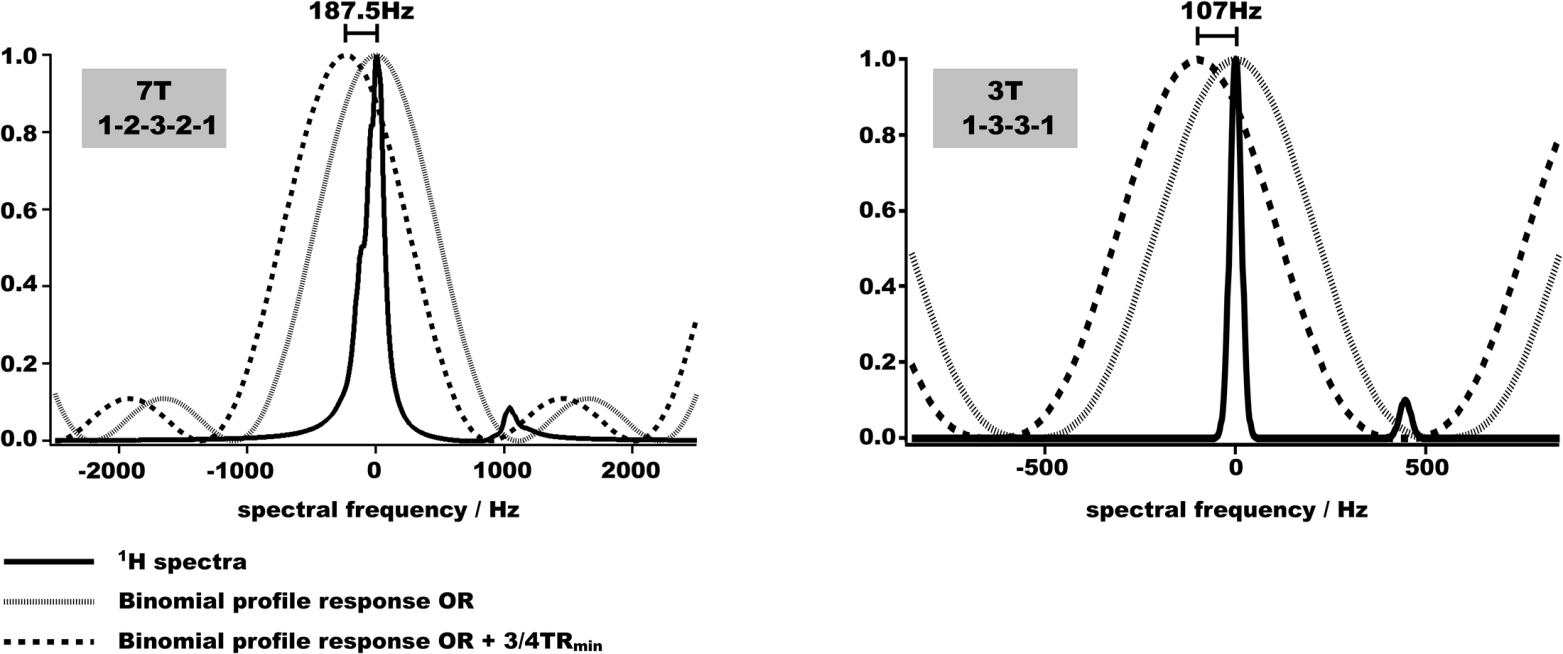
Excitation profile of the WS pulses combined with the frequency-shift method. Proton spectrums measured at 7T (water linewidth = 180Hz) or 3T (water linewidth = 40Hz). The frequency profiles of 1-2-3-2-1 or 1-3-3-1 binomial pulses are overlaid to show the selectivity of the frequency excitations. In these cases, the binomial pulses were centered on the water frequency (grey lines). The binomial profiles applied when their excitations are shifted by (3/4TR) are also overlaid (dotted lines) to demonstrate that the selectivity of excitation is preserved.

Fig 1 shows two 1H spectrums: one acquired experimentally at 7T and one simulated using the average Full Width at Half Maximum (FWHM) of the proton peak measured over several MSK experiments at 3T. On both spectrums, the main and the small peaks represent the water and the fat protons, respectively. When the excitation frequencies of the binomial pulses are centered on the water resonance, the excitation band is broad enough to correctly excite water protons even in a case of poor field homogeneities (as an example, FWHM measured on mouse abdomen at 7T was ≈190Hz). In parallel, the non-excitation band located between 840Hz and 1490Hz would generate an excitation of the fat protons lower than 4% at 7T. At 3T, this non-excitation band covering frequencies from 371Hz to 703 Hz would excite less than 1% of fat protons.

### Combination of binomial pulses and banding artifact correction

Then, these binomial pulses were tested in combination with the “Sum-Of-Square” (SOS) technique, in order to suppress banding artifacts. The SOS reconstructions were performed off-line using IgorPro (Wavemetrics, Lake Oswego, OR), by applying the square root of the sum of multiple squared images [2,5]. To do so, four WS-bSSFP images needed to be acquired at four different offset RF frequencies that are multiples of 1/4TR: On Resonance (OR); OR+(1/4TR); OR+(1/2TR); OR+(3/4TR). Thus, the frequency of the binomial pulse had to be shifted to a maximum value of (3/4TR). In order to evaluate the influence of this frequency shift on the selectivity of the binomial pulse, only the (3/4TR) case is shown. In the case of a minimum TR of 4ms at 7T and 7ms at 3T, the binomial pulses applied at frequency shifts of 187.5Hz and 107Hz, respectively, yields 87% and 85% of the RF amplitude at water proton frequency. Furthermore, fat suppression remains efficient: in that case, 2.9% and 4.7% of fat protons remains excited, respectively. In certain cases, only two offsets were acquired limiting the frequency shift of the excitation to OR+(1/2TR). Consequently, the non-excitation of the fat protons would be less affected.

### Banding artifact correction simulations

Finally, the influence of the nutation angle on banding artifacts and signals of muscle, blood and adipose tissue was also simulated for a typical TR value (7ms).

Simulations based on equations from Leupold et al and Freeman et al [12,24] demonstrated that the intensity of bandings (reflected by the successive drops of signal) in individual images acquired at a given offset frequency increases as the nutation angle becomes lower (S1 Fig). On the contrary, increasing the nutation angle can induce some limitations, like a low SNR from tissues other than fluids [25]. A nutation angle of 30° was theoretically the best compromise (i) to generate bSSFP images free of banding artifacts for all tissues, and (ii) to limit SAR deposition (SAR was inferior to 1W/kg for every experiments performed at 3T).

## Materials and Methods

### Ethics Statement

All experimental procedures were approved by the Animal Care and Use Institutional ethics committee “Comité d'éthique pour l'experimentation animale Bordeaux” of Bordeaux, France (approval n°5012032-A).

For human imaging, the study was approved by the local institutional review board for human subjects “Comité de protection des personnes—Sud-Ouest Outre-Mer III” (n° 2009-A01080-57). Before imaging, informed consent was signed for all human studies.

### Animal Preparation

Female C57BL/6NCrl mice (N = 12, 8 weeks old; Charles River Laboratories, L’Arbresle, France) were used. At the start of the experiments, animals weighed (mean±SD) 22±2g. The mice were housed in groups of 6 and given five days to acclimate to the housing facility. Environmental conditions were a temperature of 22°C, humidity of 60%, lighting of 620 lux (6 bulbs of 20W) and a 12:12 light:dark cycle with lights on at 08:00 and off at 20:00. Animals were housed in 396x215x172 mm cages (1285L, Techniplast, France) filled with corncob (SAFE France) and given access to mouse maintenance food (A04, SAFE, France) and water ad libitum. Environmental enrichment included bedding (TopBrick and Topwoodwhool, SAFE France), coton and craft paper (SAFE France), one red tinted hut (SAFE France), one 10x10x50 mm aspen chew block (SAFE France). During housing, animals were monitored daily for health status. In an effort to reduce animal use in the long term, some healthy animals were scanned several times during the development and the optimization of the sequence. A completed ARRIVE Guidelines checklist is included in S1 ARRIVE Checklist.

As a mouse cancer model, female BALB/cByJ (N = 6, Charles River Laboratories, L’Arbresle, France) were injected with 10^5^ mouse renal carcinoma cells (RenCa) in 25μL under the renal subcapsule, as already described [26]. No adverse events were observed.

### MRI Materials

#### 7T MRI

The MR experiments were carried out on a small-animal horizontal 7T magnet (Bruker, Germany), using an transmit/receive birdcage coil (inner diameter: 2.5 cm, 5 cm length; sensitive volume: 35mm). Mice were anesthetized with isoflurane (1%–1.5% in air) for its low toxicity, a high control of the anesthesia during the MR experiment and a fast wake-up of the animals. The mice were positioned supine. The respiration rate was monitored using an air balloon placed on the thorax (SA Instruments, Inc., NY, USA). An automatic shimming procedure was performed before each experiment.

#### 3T MRI

A 3T scanner (Achieva, Philips, Best, The Netherlands) was used to acquire images of 10 volunteers (age range, 28–64 years; mean age, 35.5). An automatic shimming procedure was performed before each experiment.

### Pulse Sequences

#### 7T MRI

After performing a 3D bSSFP sequence (referred as “‘standard” thereafter), a 1-2-3-2-1 water-selective binomial-shaped RF pulse was inserted into the sequence to replace the usual single pulse (the modified sequence was then named WS-bSSFP). The slice selection gradient was removed. It is important to note that respiration-triggering was never applied in our study.

For both sequences and all the applications, the following parameters were identical: nutation angle (FA) = 30°; frequency-encode direction: AP; 4 offsets (SOS4). Echo time (TE) was always equal to TR/2. The other parameters are summarized in Table 1.

**Table 1:** Parameters of the different sequences used for the several small-animal applications at 7T. All the images were reconstructed from the SOS of 4 frequency offset images.

	Sequence	FOV Matrix	NEx/offset	TR rBW	Acq Time/offset	Total Acq Time
Abdominal Imaging	Standard 3D bSSFP	30x22x22mm 192x128x128	1	4.2ms 651Hz/pixel	1min08s	4min32s
	3D WS-bSSFP	30x22x22mm 192x128x128	1	4.8ms 651Hz/pixel	1min19s	5min16s
Whole-Body Imaging	Standard 3D bSSFP	35x22.5x22.5mm 256x112x112	1	4.9ms 391Hz/pixel	1min02s	4min07s
	3D WS-bSSFP	35x22.5x22.5mm 256x112x112	1	5.9ms 391Hz/pixel	1min14s	4min56s
Lymph Node Imaging	Standard 3D bSSFP	30x22x22mm 256x128x128	2	4ms 781Hz/pixel	2min11s	8min44s
	3D WS-bSSFP	30x22x22mm 256x128x128	2	4.6ms 781Hz/pixel	2min31s	10min04s
Renal Tumor Imaging	Standard 3D bSSFP	30x22.5x25mm 192x142x160	2	4.1ms 651Hz/pixel	3min06s	12min24s
	3D WS-bSSFP	30x22.5x25mm 192x142x160	2	4.6ms 651Hz/pixel	3min29s	13min56s

#### 3T MRI

Human knee imaging was performed using a knee coil (8 channels; sensitive volume: 22cm). For ankle imaging, both ankles were positioned into a head coil (8 channels). Images of the legs of volunteers were acquired using an abdominal surface coil (16 channels). Brain images were acquired using a head coil (8 channels).

After performing a standard bSSFP sequence, a 1-3-3-1 water-frequency-selective binomial-shaped water excitation RF pulse was inserted into a standard bSSFP sequence to replace the usual single pulse (the modified sequence was called WS-bSSFP). The slice selection gradient was removed.

For both sequences and all the applications, the following parameters were identical: FA = 30°; frequency-encode direction: AP; TR = 7ms; rBW = 754/8Hz/pixel; Partial Fourier = 80%; NEx = 1; zero-filling reconstruction. Echo time (TE) was always equal to TR/2. The other parameters are summarized in Table 2.

**Table 2:** Parameters of the different sequences used for the several human applications at 3T.

	Sequence	FOV Matrix	Offset	SENSE factor	Acq Time/offset	Total Acq Time
Knee Imaging	Standard 3D bSSFP	160x160x140mm 276x263x140	1	1	3min26s	-
	3D WS-bSSFP	160x160x140mm 276x263x140	2	1	3min26s	6min52s
	3D WS-bSSFP	160x160x140mm 276x263x140	4	1	3min26s	13min44s
	3D WS-bSSFP	160x160x140mm 276x263x140	4	2	1min43s	6min52s
	3D WS-bSSFP	160x160x140mm 276x263x140	2	4	52s	1min43s
Ankle Imaging	Standard 3D bSSFP	160x160x160mm 276x263x160	1	1	3min56s	-
	3D WS-bSSFP	160x160x160mm 276x263x160	4	2	1min57s	7min48s
Leg Imaging	Standard 3D bSSFP	350x400x200mm 228x216x125	1	1	2min31s	-
	3D WS-bSSFP	350x400x200mm 228x216x125	4	2	1min15s	5min
Head Imaging	Standard 3D bSSFP	250x235x180mm 240x230x120	1	1	2min35s	-
	3D WS-bSSFP	250x235x180mm 240x230x120	4	2	1min17s	5min8s

For knee imaging, the 3D WS-bSSFP sequence was acquired either with SENSE factor 2 in combination with 4 frequency offsets or with a SENSE factor of 4 in combination with 2 frequency offsets. For the other applications, SOS 4 was used.

The SOS reconstruction was performed after the SENSE acquisitions.

### Image analysis

All the data were analyzed using IgorPro (Wavemetrics, Lake Oswego, OR).

On small-animal images, signal of subcutaneous fat, kidney cortex (mentioned as “kidney”) and leg muscles have been measured by drawing regions-of-interest (ROI) in these structures and the standard deviation of the noise was measured by placing a ROI outside of the mouse body. The SNR was calculated as the ratio between the signal of a ROI and the standard deviation of the noise. The locations of the ROI were maintained on the different offset images, so that alteration of SNR due to the presence or not of banding artifacts was taken into account.

In human images, ROIs were placed on subcutaneous fat, bone marrow, muscle, articular cartilage and synovial fluid. Then, the signals from these structures were divided by the standard deviation of the noise that was measured from a ROI placed outside of the knee.

Contrast-to-noise ratios (CNR) were calculated as the difference between SNR of the previously mentioned structures.

The experimental unit was a single animal or volunteer.

### Statistical Analysis

For small-animal imaging, the mean SNR ± standard deviation of fat, muscle and kidney cortex calculated for each mouse were averaged between the different mice (independent experiments performed at different days) and reported in Table 3.

**Table 3:** SNR of mouse sub-cutaneous fat tissue, kidney and limb muscle in standard bSSFP and WS-bSSFP images at 7T.

Sequence	Fat	Kidney	Muscle
**Standard SOS4**	87.8 ± 9.8	30.3 ± 13.3	19.9 ± 5.8
**WS OR**	4.4 ± 1 *	22.9 ± 5.5	16.9 ± 0.4
**WS OR + 166.7 Hz**	6.7 ± 0.2 *	24.3 ± 2.7	17.1 ± 1.6
**WS SOS4**	13.9 ± 1.4 *	38 ± 8.7	29.4 ± 4.7

The values obtained when the frequency excitation was shifted by 166.7Hz (corresponding to 3/4TR shift with TR = 4.5ms) or not (OR) and for SOS4 reconstruction are shown.

* represents p<0.01 compared to fat SNR on standard images.

For human knee imaging, the mean SNR ± standard deviation of fat, bone marrow, muscle, articular cartilage and synovial fluid were averaged between volunteers who were scanned at different days and were reported in Table 4.

**Table 4:** SNR of human sub-cutaneous fat tissue, bone marrow, joint fluid, cartilage and leg muscle in standard bSSFP and WS-bSSFP images at 3T.

Sequence (AcqTime)	Fat	Bone Marrow	Synovial Fluid	Cartilage	Muscle
**Standard** (3min26s)	27.3 ± 1.4	19.4 ± 0.4	37.8 ± 3.7	12 ± 1.1	10 ± 0.2
**WS OR** (3min26s)	2.4 ± 0.7 *	1.1 ± 0.2 *	33.6 ± 2.8	12.4 ±1.5	8.7 ± 0.5
**WS OR + 107Hz** (3min26s)	1.8 ± 1.2 *	1.4 ± 0.3 *	44.1 ± 11.3	14.7 ± 1.9	11 ± 1.6
**WS-SOS2** (6min52s)	3.6 ± 1.1 *	1.7 ± 0.2 *	47.6 ± 4.1	17.7 ± 2.8	13.1 ± 1.4
**WS-SOS4** (13min44s)	5.2 ± 0.8 *	2.6 ± 0.3 *	67.5 ± 4.2	25.4 ± 3.3	19.2 ± 1.7
**WS-SOS4 + SENSE 2** (6min52s)	4.2 ± 0.3	2.5 ± 0.3	32.5 ± 1.3 °	19.7 ± 1.2 °	17.7 ± 0.3
**WS-SOS2 + SENSE 4** (1min43s)	2.3 ± 0.4 * ^†^	1.4 ± 0.1 * ^†^	25.1 ± 5.5 # ^†^	10.4 ± 1.2 # ^†^	7.9 ± 0.7 # ^†^

The values were measured when the frequency excitation was shifted by 107Hz (corresponding to (3/4TR) with TR = 7ms) or not (OR) and for SOS2 or SOS4 reconstruction. The WS-bSSFP sequence was also combined with parallel imaging (SENSE 2 or 4) to reduce acquisition time. Acquisition time is shown in brackets under the sequence name.

* represents p<0.01 compared to fat and bone marrow SNR on standard images.

° represents p<0.05 compared to SNR on WS-SOS4 images.

^#^ represents p<0.05 compared to SNR on WS-SOS2 images.

^†^ represents p<0.05 compared to SNR on WS-SOS4 SENSE2 images.

The SNR measurements measured in the different 3D bSSFP images were compared using paired Student’s t-test. P < 0.05 was considered a significant difference.

## Results

### Small Animal Imaging at 7T

**Fig 2:**
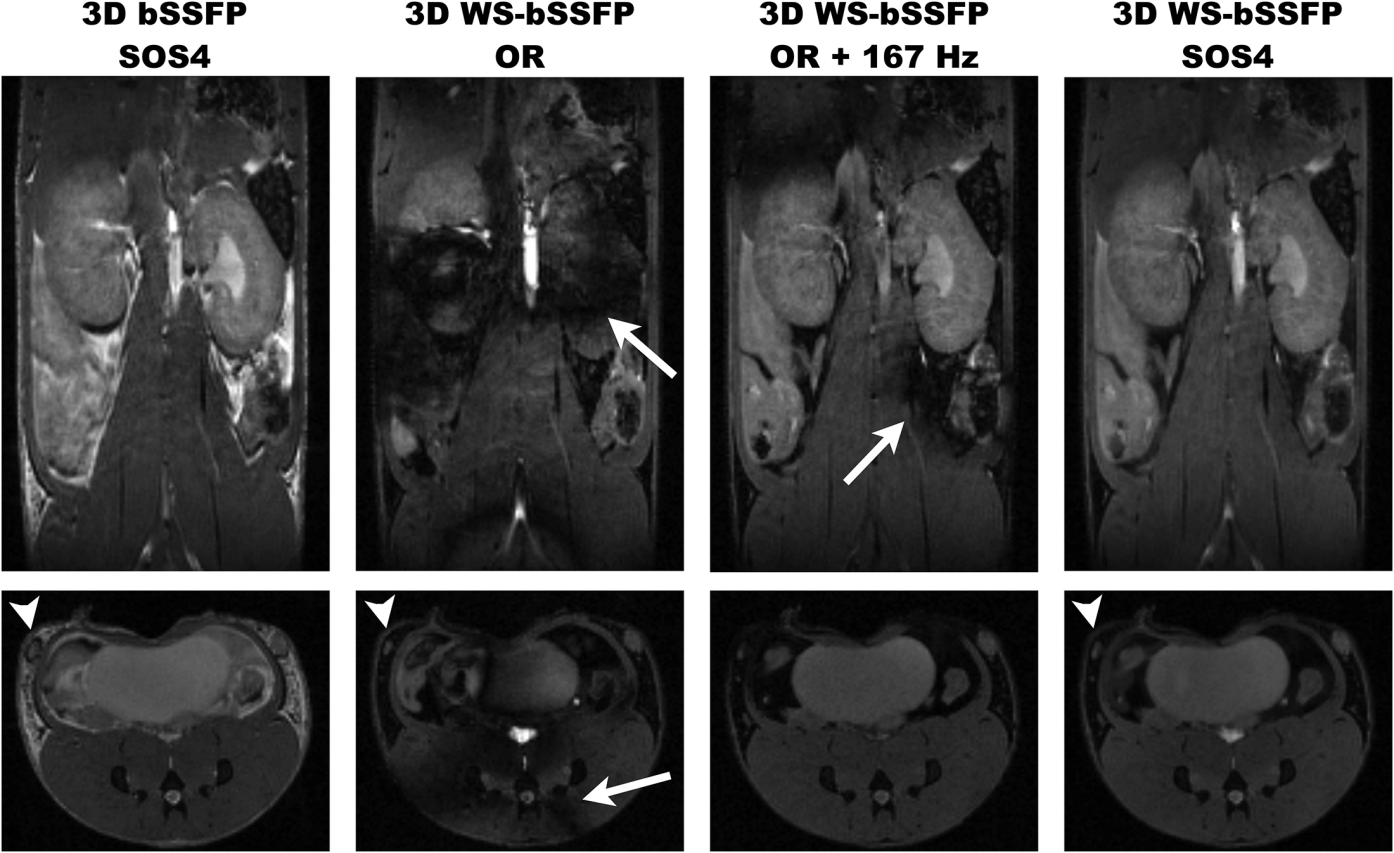
3D coronal images of mouse abdomen acquired at 7T using standard bSSFP or WS-bSSFP sequences. The images shown were either acquired On Resonance (OR), OR + (3/4TR) apart from the resonance (for TR = 4.5ms, the shift equals 166.7Hz), or summed using the SOS technique (SOS4). Arrows point at banding artifacts. Arrowheads indicate the inguinal lymph node location.

Mouse abdomen imaging was performed at 7T (Fig 2) using a standard bSSFP sequence with a conventional non-binomial RF pulse. Adipose tissues were detected as hyperintense areas (Table 3). Chemical shift artifacts were present particularly between kidneys and visceral fat and around the inguinal lymph nodes (arrowheads). Then, the 3D WS-bSSFP sequence was used. Images acquired with the smaller (0Hz) and the larger (166.7Hz) excitation frequency offsets are shown in Fig 2. In both images, the fat signal was nearly cancelled on the entire FOV (p = 0.004). The banding artifacts were broad (representing between 10 to 24 pixels width), differently located on the two images. Figure A in S2 Fig shows the multiple locations of drop of signals in the mouse kidney in function of the frequency shift. After the SOS 4 reconstruction, the kidney signal was more homogeneous. Quantitatively, the SNR of muscles affected by these signal losses was 4.8±0.5 on the OR images. After applying SOS4 reconstruction, no banding artifact could be detected on the entire 3D WS-bSSFP image (the SNR of muscle affected by banding artifacts was 31.1±6.2, returning to the mean muscle SNR of 29.4±4.7 (p = 0.017)).

In addition, as shown in Table 3, compared to the 3D bSSFP standard sequence, the fat SNR was 6.3-times lower (p = 0.007) while the SNR of other tissues like kidney and muscles were maintained on the 3D WS-bSSFP image. The CNR between kidneys and muscles was not significantly different on standard bSSFP and WS-bSSFP images (p = 0.58). Lymph nodes could then be more accurately delineated. An acquisition time of 5min16s was necessary to obtain the final SOS4 image, each frequency offset acquisition lasting 1min19s.

**Fig 3:**
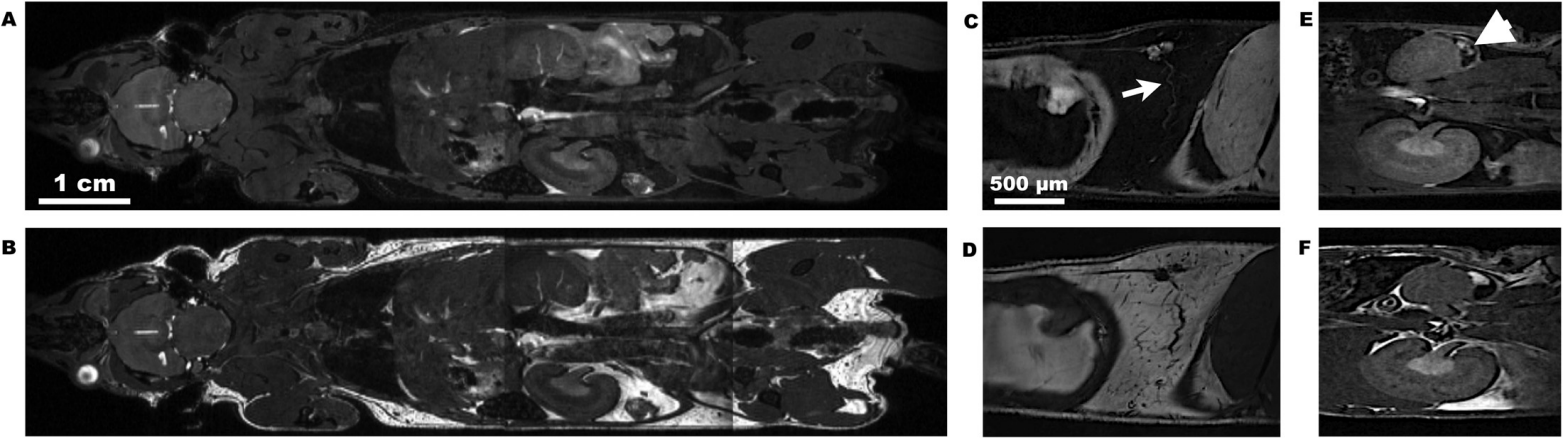
Small-animal applications of the 3D WS-bSSFP sequence at 7T. Coronal mouse whole-body images using standard bSSFP (B) and WS-bSSFP (A) sequences. Sagittal standard bSSFP (D,F) and WS-bSSFP (C,E) images showing the inguinal lymph node (C,D) and its corresponding lymphatic vessel (arrow) and a primary tumor (E,F) developing into the renal cortex (arrowhead).

The 3D WS-bSSFP sequence was then validated for several applications on small animals at 7T. Whole-body imaging was performed as shown in Fig 3A and 3B. Acquiring four 3D WS-bSSFP images allowed to visualize the entire mouse body without fat signal (Fig 3A), even in areas with poor field homogeneity (lungs, abdomen). The organs were well differentiated and fluids that still appeared as hyperintense signal on the images like cerebrospinal fluid and vessels, could be then distinguished from fat signal.

As expected, the suppression of the fat signal deleted chemical shift artifacts. The inguinal lymph nodes and their emerging vessels were thus better delineated on WS-bSSFP images than on standard bSSFP images (Fig 3C and 3D). Furthermore, tumors growing in the kidney cortex at the interface between renal tissue and fat, were accurately distinguished from visceral fat after the binomial pulse was applied. On the contrary, on standard bSSFP images, tumors can exhibit similar hypersignal than fat that could be misleading for diagnosis (Fig 3E and 3F).

### Human imaging at 3T

**Fig 4:**
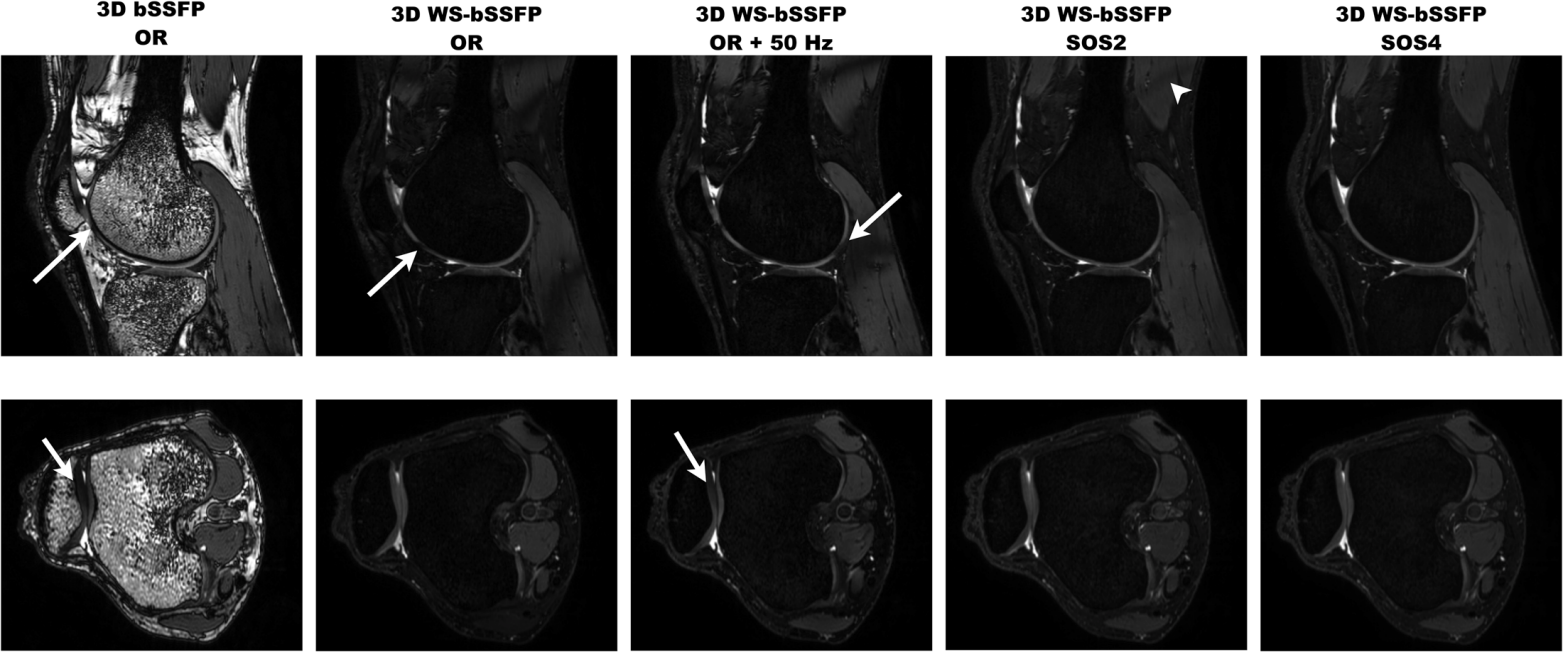
3D human knee images acquired at 3T using standard bSSFP and WS-bSSFP sequences in combination with parallel imaging. The sagittal and axial images shown were either acquired On Resonance (OR + 0Hz), OR + (3/4TR) apart from the resonance (for TR = 7ms, the shift equals 107Hz), or summed using SOS technique (SOS2 or SOS4) and in combination with parallel imaging (SENSE 4). Arrows point at banding artifacts altering cartilage measurements. The arrowhead indicates a banding artifact in the muscle at the edge of the FOV.

The 3D WS-bSSFP sequence was then applied on a clinical MRI system at 3T in order to perform human musculoskeletal imaging (Fig 4, Table 4). Images acquired using the standard bSSFP sequence without the binomial pulse exhibited bone marrow and fat with hyperintense SNR (19.4±0.4 and 27.3±1.4, respectively). The presence of the chemical shift artifacts could alter the accurate measurement of cartilage thickness. Furthermore, as the synovial fluid engendered similar signal intensity than adipose tissue, its detection was impeded. However, as soon as the binomial pulse was used, the SNR from subcutaneous fat and bone marrow drastically decreased to 2.4±0.7 (p = 7x10^-4^) and 1.1±0.2 (p = 4x10^-5^), respectively. The SNR of other tissues (muscle, cartilage, joint fluid) remained similar (Table 4), except at the location of banding artifacts. For example, SNR of muscles affected by large bandings (example on Fig 4 arrowhead) decreased from 8.7±0.5 to 2.5±0.3 on the OR images. Fat SNR was inferior to 5 on the entire knee image even though the binomial pulse frequency was shifted by 107Hz (representing the maximum offset reached at TR = 7ms), whereas the signals from the other knee structures were significantly higher (Table 4).

Finally, SOS4 reconstruction removed all banding artifacts in the entire FOV. As demonstrated in Figure B in S2 Fig, the multiple drop of signals in the cartilage occurring in the images acquired at the different frequency shifts were not detectable anymore on the SOS 4 images. The SNR from muscle areas affected by banding artifacts increased to 22±2.1, reaching the SNR value of the whole mean muscle (19.2±1.7). The CNR between cartilage and synovial fluid was not significantly different on the images acquired with the standard bSSFP and the WS-bSSFP sequences (p = 0.08).

However, acquisition time during clinical exam is critical. To reduce this parameter, parallel imaging was applied using acceleration factors of 2 or 4 (SENSE 2 or 4).

After applying parallel imaging, the signals in the areas of subcutaneous fat and bone marrow was still extremely low (Table 4). As expected, the SNR of the cartilage and the synovial fluid decreased significantly on SENSE 2 and 4 images compared to images performed without parallel imaging. However, the CNR between these two regions was maintained high, at 15.8±1.6 and 15.1±4.5 on SENSE 2 and 4 images, respectively. Furthermore, contrasts between cartilage and bone marrow (15.7±1.1 and 9±1.3 on SENSE 2 and 4, respectively) and between joint fluid and subcutaneous fat (27.4±1.7 and 23±5.6 on SENSE 2 and 4, respectively) were still high enough to identify these structures easily.

Thus, knee images acquired with 3D WS-bSSFP sequence could be generated with a high resolution (580x608μm) within 2 minutes only, using a SENSE factor of 4. Nevertheless, as SNR and CNR were significantly higher on WS-bSSFP SENSE 2 SOS4 than on WS-bSSFP SENSE 4 SOS2, following human images were performed with the WS-bSSFP SENSE 2 SOS4 sequence to ensure that no banding artifact will be present on the images. This method allowed to easily detect knee arthritis in one of the volunteer, demonstrating the suitability of the 3D WS-bSSFP sequence for MSK imaging.

**Fig 5:**
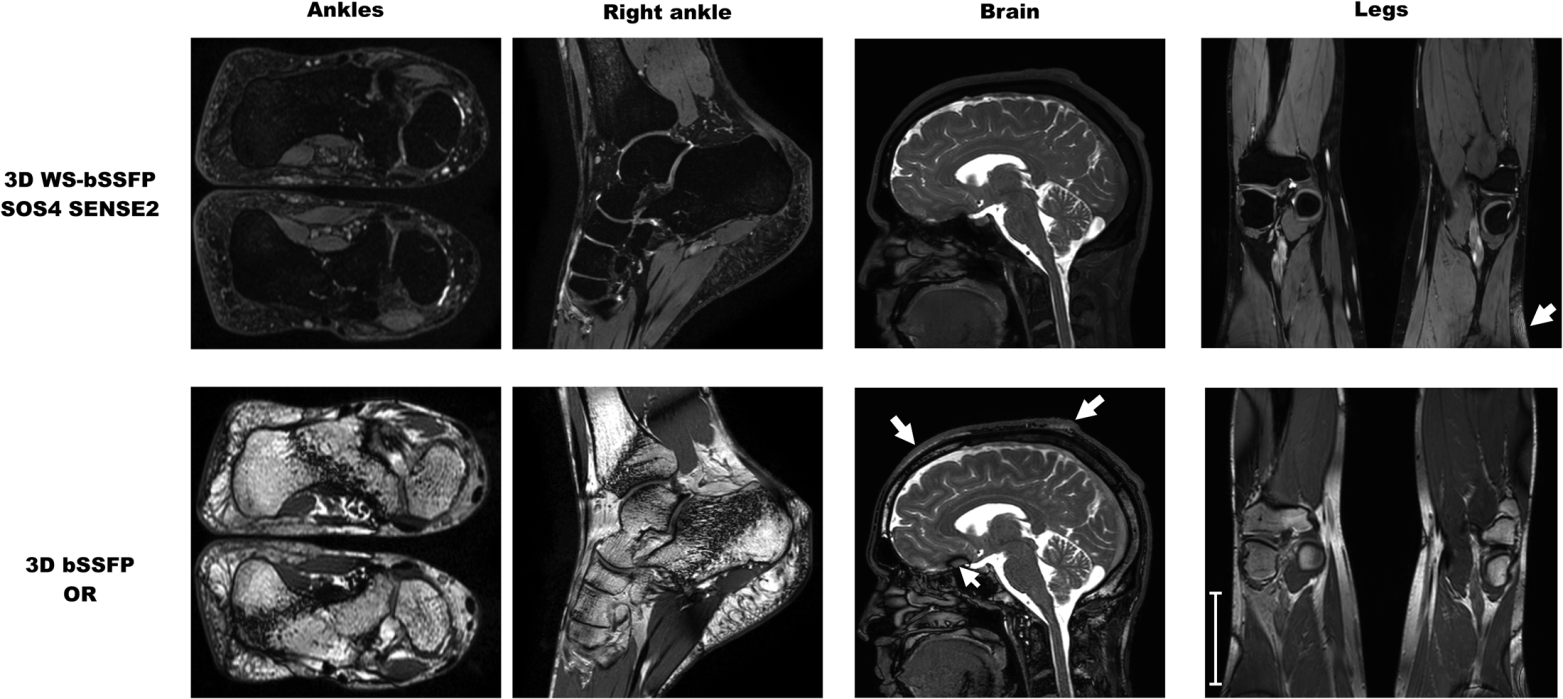
Human applications of WS-bSSFP sequences at 3T. 3D human ankle images, brain and legs of healthy volunteers using standard bSSFP and WS-bSSFP sequences with a SENSE factor of 2. The arrow in the leg WS-bSSFP image indicates remaining fat signal at the edge of the FOV, whereas the ones on the head bSSFP images point at subcutaneous fat and a banding artifact is shown by the arrowhead. Scale bar represents 10cm.

3D standard bSSFP and WS-bSSFP images of both ankles of a healthy volunteer are shown in Fig 5. These images demonstrated the robustness of the technique developed in this study even in areas of the body particularly affected by inhomogeneities in the magnetic field that could alter the efficiency of the binomial pulse and the banding artifact correction. In addition, 3D WS-bSSFP images were also obtained on large FOV (between 25 and 35cm) on brains and legs of healthy volunteers (Fig 5). No banding artifact was detected and the water frequency-selection of the binomial pulse was efficient, even if some fat signal remained at the edges of the large FOV due to the limit of the B0 field homogeneity of the magnet (arrow on the leg images).

## Discussion

This study demonstrated that a binomial water-selective pulse could be combined with bSSFP sequence in order to create fat-free 3D images at high magnetic fields. Nowadays, more studies are performed at high or ultra-high magnetic fields. However, in small animal and human imaging at 7T and at 3T, numerous banding artifacts occur due to the use of the bSSFP sequence, particularly in areas with poor magnetic field homogeneity. These artifacts were present even when short TR values were used at 3T [17]. Nevertheless, as already demonstrated, the method of frequency offset combined with SOS reconstruction allows for an efficient correction of banding artifacts. It is shown here that a highly water-selective binomial pulse can be combined with this method to properly minimize fat signal and banding artifact. To do this, the non-excitation band generated by the binomial pulse needed to be broad enough to not excite fat protons in a wide range of frequencies. In parallel, the water pass band had to be large enough to always excite water protons even after the shift of excitation frequencies necessary to suppress banding artifacts.

The most efficient way to prevent exciting fat protons was to employ 4 or 5 sub-pulses binomial patterns (depending on the MRI manufacturer). It has been observed that, if less selective binomial pulses are used, fat signal suppression is less optimal [18], particularly when the excitation and resonance frequencies are far apart and when the magnetic field homogeneity is poor. However, the insertion of several sub-pulses induced an increase in the minimum TE of approximately 500μs or 1ms at 7T and 3T, respectively and could limit the use of large nutation angle for SAR reasons. This could be an issue for angiography studies. However, in this study, this was not a concern as the contrast generated by the bSSFP sequence was high enough to detect kidney tumors in mice and cartilage in human knee and ankle joints. The CNR values obtained on WS-bSSFP images after SOS reconstruction were not significantly different from the ones obtained on standard bSSFP images. This result is in agreement with a previous study comparing standard bSSFP images and either complex-summed or SOS reconstructed images [2]. Due to the very low SNR of adipose tissue, developing metastases could be easily detected as hyperintense areas within lymph nodes and bone marrow due to the T2/T1 contrast generated by the bSSFP sequence. This can be an asset for performing an early diagnosis of cancer spreading.

Parallel imaging, commonly performed in clinic, did not alter significantly these contrast values (Table 4). CNR between cartilage and fluid was higher compared to those found in clinical practice [27]. For MSK imaging, WS-bSSFP sequence displayed greater benefits compared to the default WS-mGRE sequence due to higher qualitative detection of cartilage and synovial fluid (with other knee structures) in shorter acquisition times (data not shown). The 3D human knee images were acquired in 1min43sec, while less than 8min were necessary to generate 500μm-isotropic images of both ankles. Furthermore, imaging large FOV was also possible as illustrated by leg and brain images. The ability to produce 3D WS-bSSFP images free of fat signal and without intense banding artifacts in such a reduced time highlights the potential of this technique to be transferred to clinical practice. There is a growing interest in using 3D imaging protocols on humans particularly with the bSSFP sequence due to its well-known features (high SNR in fast acquisition time), as shown by a very recent study proposing a 2-point Dixon 3D bSSFP sequence combined with Complex-Sum reconstruction [23]. Only three studies were already performed to both suppress banding artifacts and reduce fat signal. These studies used phase-detection bSSFP imaging. This alternative method is robust at 3T and at 1.5T, but induced restrictions in TE values, some being impractical to achieve as the magnetic field strength increases. Furthermore, the reconstruction time could be as long as 2 hours depending on the matrix size and the parallel imaging configuration used. In addition, this method has already been shown to be inherently sensitive to partial-volume effect and phase induced by spatially-varying coil sensitivity [13], that would introduce errors in fat/water separation, limiting its application for quantitative bSSFP imaging [23]. In comparison, our magnitude-based method with similar acquisition times as these previous papers, induced similar image quality with no need of acquiring a field-map or generating algorithms [22] and no restriction in TE and flip angle values.

In our case, the minimum TE could be kept very short (5ms) or increased depending on the application. This could be of great interest to improve the T2 or T2* weighting of the sequence in order to either detect iron-labeled cells or iron-loaded organs (like the liver and spleen), due to a higher iron sensitivity with long TE/TR, or to detect cerebral microbleeds [28] or vascular insufficiencies [29] on susceptibility-weighted images.

One of the limitations of the present method is the use of non-spatially selective pulses. It was thus necessary to set FOVs that enclosed the entire areas covered by the coils, increasing acquisition time. Nevertheless, spectrally-selective and spatially-selective (SPSP) pulses have already been developed. As demonstrated by Schick at 1T [30], the spectral and spatial profiles of SPSP pulses illustrated that they could be compatible with both a spatial selective imaging and frequency shifting for banding artifact suppression even at high magnetic fields. However, only few studies have inserted them into a bSSFP sequence. Cartilage volume was measured on high in-plane resolution 3D bSSFP images at 3T, but banding artifacts were observed at the edges of the tissue [31]. Yuan et al applied a 5 SPSP sub-pulse excitation train to generate 2D abdominal images at 3T [18]. To limit the presence of banding artifacts, a compromise between the thickness of the 2D slices and the length of the pulse (influencing the TR value) was encountered. This affected the homogeneity of the fat or water suppression on the images. Nevertheless, techniques that use parallel excitation would limit the increase in TE and manage SAR [32].

Finally, the main limitation of our technique is the necessity to acquire 2 or 4 WS-bSSFP images to sum them using the SOS technique. One way that could be explored would involve improvements in post-processing techniques [33]. Although they have never been applied to MRI yet, they could potentially remove banding artifacts while acquiring solely one bSSFP image. On the other side, in small animals, multiple acquisition is not a major issue, as several NEx have to be performed to increase SNR due to high spatial resolution. However, acquisition time is crucial in clinical settings. The present method was perfectly compatible with parallel imaging allowing to obtain 3D knee images with both high in-plane resolution and thin partitions (1mm in our case) in 1min43s. This result could even be improved by the use of 16-channel coils that are currently available in clinical systems. As already demonstrated, radial ATR-bSSFP sequence also allowed for the acquisition of 0.3mm isotropic knee images in 8min at 3T [34]. The method described here can be considered as a surrogate to give more flexibility to TR values and limit post-processing. The frequency-selective technique is implemented on most clinical and preclinical scanners and thus could be used in both human and small-animal MR studies. Furthermore, the present technique needs simple SOS reconstruction post-processing, contrarily to the post-processing needed in Dixon methods or multiple-echo methods [35,36,37,38]. In addition, TE/TR and nutation angle values are not excessively limited contrarily to the technique proposed by Quist et al [22] whose efficiency is limited by the use of nutation angles greater than 70° or the phase-correction technique [39] that can generate unwanted partial-volume artifacts.

When developing such MR sequences to evaluate its applications in small-animals and humans, a large reduction in animal use is very likely to produce positive evidence to support the hypothesis. This report constitutes the documentation of the experimental design as a foundation for future use as a sequence for whole-body small-animal imaging and human MSK imaging.

In conclusion, this technique is robust enough to be efficiently used on small animals and on humans at high magnetic fields. Fat SNR was minimized within large FOV and banding artifacts were efficiently suppressed in areas with low field homogeneity. Due to the contrast and high SNR achieved using this sequence, 3D WS-bSSFP could be applied in several applications in high-resolution anatomical imaging, both in animals and humans.

## Supporting Information

S1 ARRIVE Checklist Animal Research: Reporting In Vivo Experiments. (PDF)

S1 FigSimulation of banding artifacts correction.The simulated frequency responses with bSSFP sequence was calculated as the magnitude of the magnetization (M_xy_) with:
$$M_{xy}=\;M_{x}+\;iM_{y}$$(1)
$$M_{x}=\;M_{0}\left(E_{1}-\;1\right)E_{2}sin\alpha sin\theta /D$$(2)
$$M_{y}=\;M_{0}\left(1\;-\;E_{1}\right)sin\alpha \left(1\;+\;E_{2}cos\theta \right)/D$$(3)
$$E_{1}=\;exp\;\left(-TR/T_{1}\right)$$(4)
$$E_{2}=\;exp\;\left(-TR/T_{2}\right)$$(5)
$$D\;=\;\left(1\;-\;E_{1}cos\alpha \right)/\left(1\;+\;E_{2}cos\theta \right)\;-\;E_{2}\left(E_{1}-\;cos\alpha \right)\left(E_{2}+\;cos\theta \right)$$(6)
with α the nutation angle of the RF pulse and θ the dephasing during TR.The frequency response was simulated for M_xy_ (OR), M_xy_ (OR+1x(1/4TR), M_xy_ (OR+2x(1/4TR) and M_xy_ (OR+3x(1/4TR). Frequency profiles for experiments reconstructed after applying the SOS on 2 or 4 acquisitions were calculated as follow:
$$M_{\left(SOS2\right)}=\;sqrt\;(M^{2}{}_{xy}\left(OR\right)\;+\;M^{2}{}_{xy}\left(OR+2x\left(1/4TR\right)\right)$$
$$M_{\left(SOS4\right)}=\;sqrt\;(M^{2}{}_{xy}\left(OR\right)\;+\;M^{2}{}_{xy}(OR+1x\left(1/4TR\right)\;+\;M^{2}{}_{xy}(OR+2x\left(1/4TR\right)\;+\;M^{2}{}_{xy}\left(OR+3x\left(1/4TR\right)\right)$$
The signal was reported as a function of the nutation angle (20°, 30° and 60°) with a typical TR of 7ms. Three sets of relaxation times (T1 and T2) were used: 350ms and 40ms; 1250ms and 50ms; 1250ms and 400ms. These values are similar to fat, muscle and blood relaxation values found in the literature. The signals after SOS2 and SOS4 reconstructions are represented in the graphs in the left and right columns, respectively.(TIF)

S2 Fig WS-bSSFP signal profiles across the mouse right kidney at 7T (A) and along the cartilage of a volunteer at 3T (B). The profiles were measured along the pink lines. The arrows point at drops of signal due to the presence of banding artifacts that occur at multiple locations in the images. Each curves correspond to the signal across the tissue of interest on WS-bSSFP images acquired either On Resonance (OR), with frequency shifts of 1/4TR, 1/2TR or 3/4TR, and after the SOS 4 reconstruction.
(TIF)

## Abbreviations

WS: Water-Selective
bSSFP: balanced Steady State Free Precession
TE: Echo Time
TR: Repetition Time
RF: Radiofrequency
SOS: Sum-Of-Square
SOS2: Sum-Of-Square performed with 2 opposite phase offsets
SOS4: Sum-Of-Square performed with 4 opposite phase offsets
SAR: Specific Absorption Rate
OR: On Resonance
SENSE: Sensitivity Encoding
SENSE2: Sensitivity Encoding performed with a factor 2
SENSE4: Sensitivity Encoding performed with a factor 4
T1, T2: longitudinal and transversal relaxation times
FOV: Field-Of-View
