# Supplementary figures and images for: Water Selective Imaging and bSSFP Banding Artifact Correction in Humans and Small Animals at 3T and 7T, Respectively
Source: PLoS One. 2015 Oct 1;10(10):e0139249. doi: 10.1371/journal.pone.0139249 (PMC4591352; doi:10.1371/journal.pone.0139249)

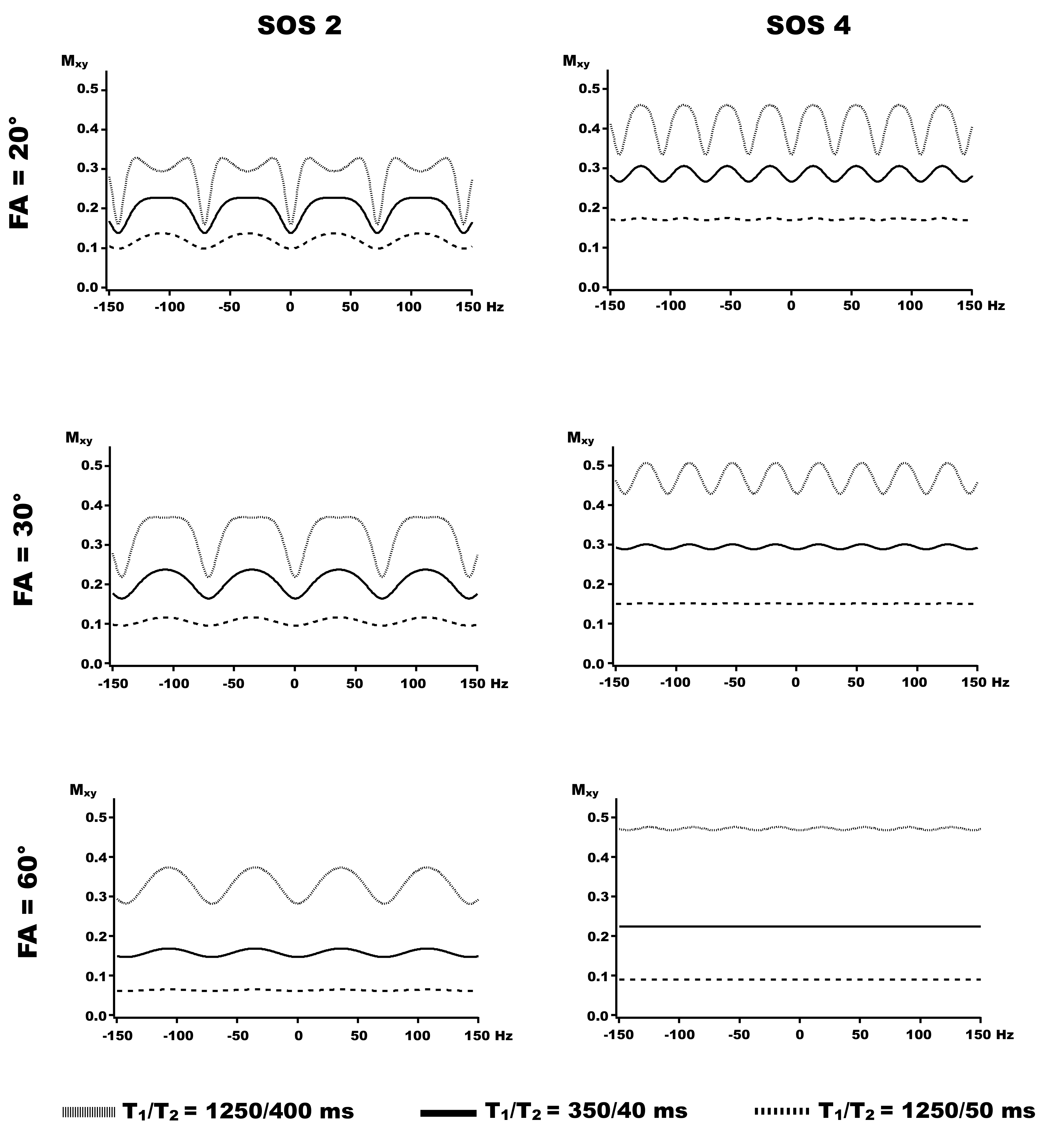

Supplement: S1 Fig — The simulated frequency responses with bSSFP sequence was calculated as the magnitude of the magnetization (Mxy) with: Mxy= Mx+ iMy(1) Mx= M0(E1− 1)E2sinαsinθ/D(2) My= M0(1 − E1)sinα(1 + E2cosθ)/D(3) E1= exp (−TR/T1)(4) E2= exp (−TR/T2)(5) D = (1 − E1cosα)/(1 + E2cosθ) − E2(E1− cosα)(E2+ cosθ)(6) with α the nutation angle of the RF pulse and θ the dephasing during TR. The frequency response was simulated for Mxy (OR), Mxy (OR+1x(1/4TR), Mxy (OR+2x(1/4TR) and Mxy (OR+3x(1/4TR). Frequency profiles for experiments reconstructed after applying the SOS on 2 or 4 acquisitions were calculated as follow: M(SOS2)= sqrt (M2xy(OR) + M2xy(OR+2x(1/4TR)) M(SOS4)= sqrt (M2xy(OR) + M2xy(OR+1x(1/4TR) + M2xy(OR+2x(1/4TR) + M2xy(OR+3x(1/4TR)) The signal was reported as a function of the nutation angle (20°, 30° and 60°) with a typical TR of 7ms. Three sets of relaxation times (T1 and T2) were used: 350ms and 40ms; 1250ms and 50ms; 1250ms and 400ms. These values are similar to fat, muscle and blood relaxation values found in the literature. The signals after SOS2 and SOS4 reconstructions are represented in the graphs in the left and right columns, respectively. (TIF) [file pone.0139249.s002.tif]

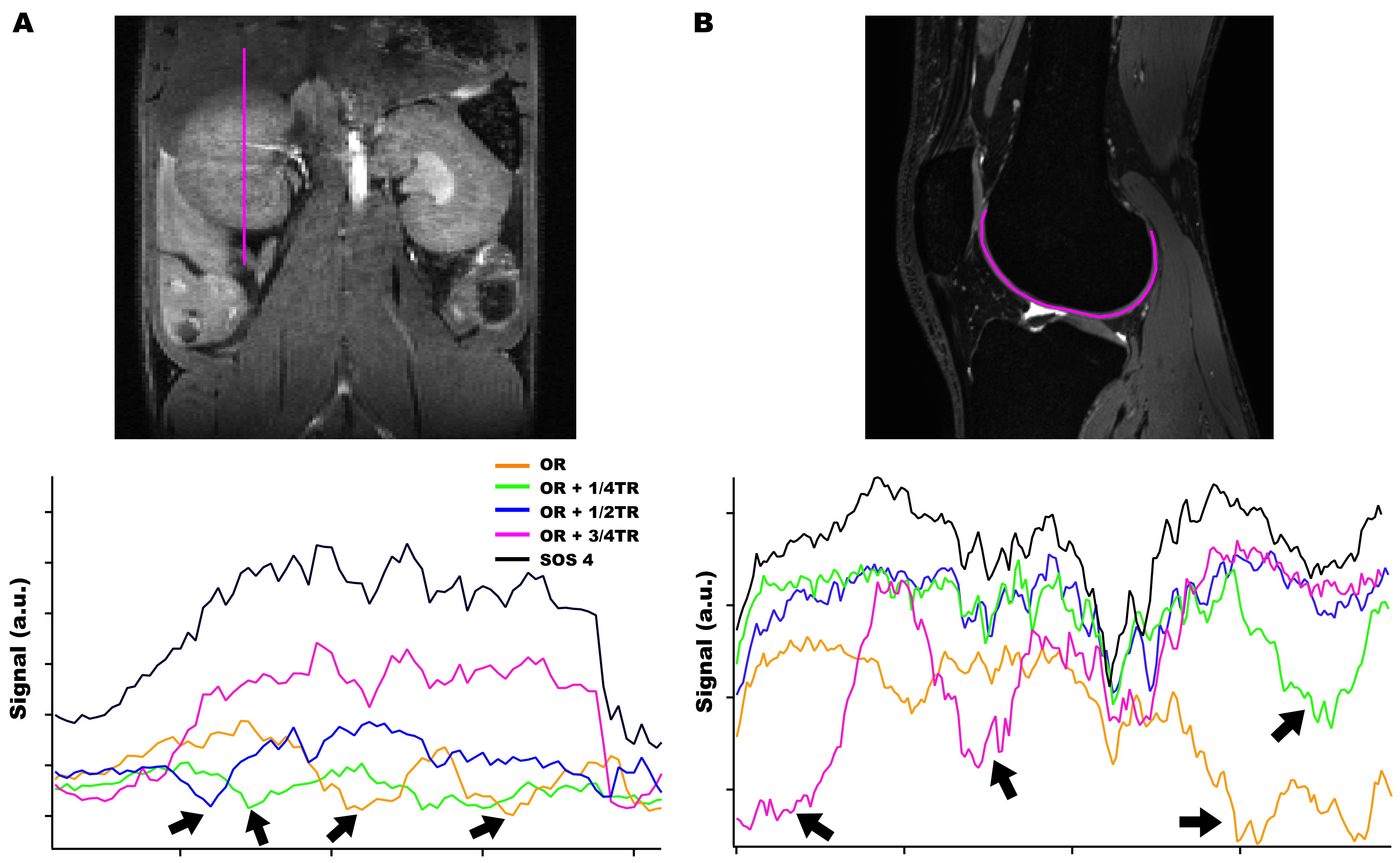

Supplement: S2 Fig — The profiles were measured along the pink lines. The arrows point at drops of signal due to the presence of banding artifacts that occur at multiple locations in the images. Each curves correspond to the signal across the tissue of interest on WS-bSSFP images acquired either On Resonance (OR), with frequency shifts of 1/4TR, 1/2TR or 3/4TR, and after the SOS 4 reconstruction. (TIF) [file pone.0139249.s003.tif]
